# PAK1 regulates RUFY3-mediated gastric cancer cell migration and invasion

**DOI:** 10.1038/cddis.2015.50

**Published:** 2015-03-12

**Authors:** G Wang, Q Zhang, Y Song, X Wang, Q Guo, J Zhang, J Li, Y Han, Z Miao, F Li

**Affiliations:** 1 Department of Cell Biology, Key Laboratory of Cell Biology, Ministry of Public Health and Key Laboratory of Medical Cell Biology, Ministry of Education, China Medical University, Shenyang 110001, China; 2 Department of Pathology, China Medical University, Shenyang 110001, China; 3 Department of Surgical Oncology and General Surgery, First Hospital of China Medical University, Shenyang, China

## Abstract

Actin protrusion at the cell periphery is central to the formation of invadopodia during tumor cell migration and invasion. Although RUFY3 (RUN and FYVE domain containing 3)/SINGAR1 (single axon-related1)/RIPX (Rap2 interacting protein X) has an important role in neuronal development, its pathophysiologic role and relevance to cancer are still largely unknown. The purpose of this study was to elucidate the molecular mechanisms by which RUFY3 involves in gastric cancer cell migration and invasion. Here, our data show that overexpression of RUFY3 leads to the formation of F-actin-enriched protrusive structures at the cell periphery and induces gastric cancer cell migration. Furthermore, P21-activated kinase-1 (PAK1) interacts with RUFY3, and promotes RUFY3 expression and RUFY3-induced gastric cancer cell migration; inhibition of PAK1 attenuates RUFY3-induced SGC-7901 cell migration and invasion. Importantly, we found that the inhibitory effect of cell migration and invasion is significantly enhanced by knockdown of both PAK1 and RUFY3 compared with knockdown of RUFY3 alone or PAK1 alone. Strikingly, we found significant upregulation of RUFY3 in gastric cancer samples with invasive carcinoma at pathologic TNM III and TNM IV stages, compared with their non-tumor counterparts. Moreover, an obvious positive correlation was observed between the protein expression of RUFY3 and PAK1 in 40 pairs of gastric cancer samples. Therefore, these findings provide important evidence that PAK1 can positively regulate RUFY3 expression, which contribute to the metastatic potential of gastric cancer cells, maybe blocking PAK1-RUFY3 signaling would become a potential metastasis therapeutic strategy for gastric cancer.

Gastric cancer is the second leading cause of cancer-related death worldwide, and the underlying molecular mechanisms responsible for gastric cancer metastasis are needed to be elucidated. Invasion of tumor cells is the key step in determining the aggressive phenotype of human cancers and compose the paramount causes of cancer deaths.^1^ The motility and invasion of cancer cell participates in a complex and integrated series of events that are primarily controlled by the regulation and reorganization of the actin cytoskeleton.^1, 2^ Regulation of actin polymerization is responsible for the formation of protrusive structures that are essential for tumor cell movement and invasion, including filopodia, lamellipodia and invadopodia.^3^ To improve the survival rate of cancer patients, it is of practical significance to investigate the proteins governing metastasis and to identify novel prognostic markers and therapeutic targets.

Human RUFY3 (RUN and FYVE domain containing 3), also known as RIPX (Rap2 interacting protein X) or Singar1 (single axon-related1), is a 469-amino-acid protein and is the highly expressed in brain tissue. The N-terminal region of RUFY3 and its homologs, including RPIP8^4^ and RPIP9,^5^ contains the RUN domain, which can interact with Rap2^4, 5, 6^ and Rab.^7, 8^ The crystal structures indicate that RUFY3 contains a RUN domain^9^ and two coiled-coil domains.^10^ Several proteins containing RUN domain have been shown to be involved in Ras-like GTPase signaling^11^ and Rab-mediated membrane trafficking.^12, 13, 14, 15, 16^ RUFY3 is thought to localize in growth cones and have a role in neuronal development by suppressing the formation of surplus axons to maintain optimal neuronal polarity.^17, 18^ However, up to date, its pathophysiologic role and relevance to cancer metastasis are still unexplored.

The human RUFY3 was identified by a yeast two-hybrid assay using P21-activated kinase-1 (PAK1) as a bait protein in our studies. The PAKs, a family of serine/threonine protein kinases, have pivotal roles in cytoskeletal reorganization,^19^ survival,^20^ motility^21, 22^ and tumorigenesis.^23^ There has been mounting evidence that PAK1 is tightly related to the progression and metastasis of cancer and may become a promising diagnostic and therapeutic target for cancer.^24, 25^ For example, elevated PAK1 expression is correlated with cancer progression and lymph node metastases in gastric cancer tissues.^26, 27^ Therefore, it is worthwhile to study the novel binding partners of PAK1.

In this study, we report that RUFY3 localizes in F-actin-enriched invadopodia and induces the formation of protrusive structures. Importantly, we found that the overexpression of RUFY3 promotes gastric cancer cell migration and invasion. Furthermore, we showed that PAK1 can affect RUFY3-mediated gastric cancer cell migration and invasion by regulating its expression. In gastric cancer samples, we showed a positive relationship between PAK1 and RUFY3, and that increased expression of RUFY3 is positively correlated with clinical gastric cancer samples. This report is the first investigation focused on exploring the role of RUFY3 in cancer cells and the relationship between PAK1 and RUFY3.

## Results

### Overexpression of RUFY3 leads to the formation of F-actin-enriched protrusion at the cell periphery

**Figure 1:**
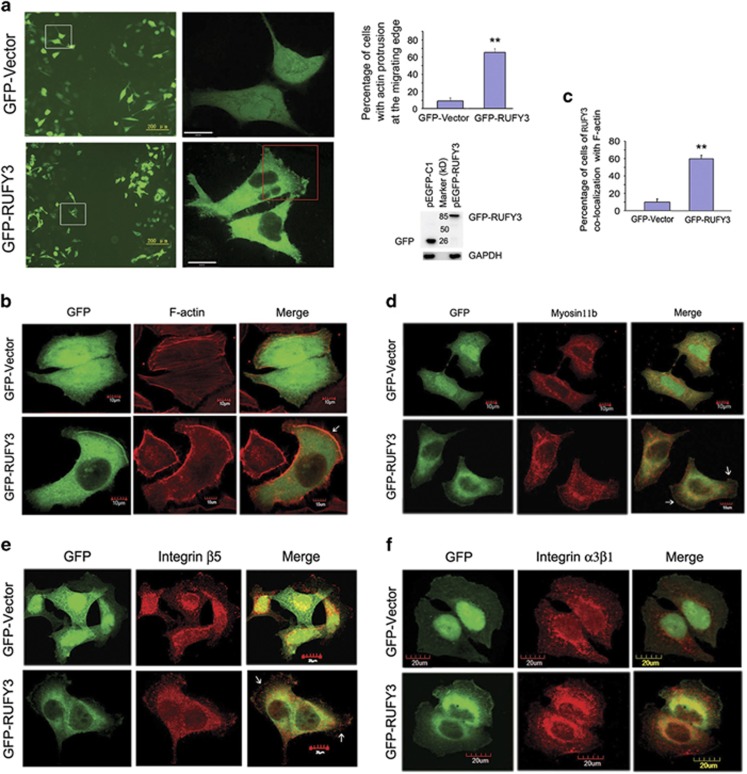
Overexpression of RUFY3 induces the formation of F-actin-enriched protrusion at the cell periphery. (**a**) GFP-RUFY3 localizes in F-actin-enriched invadopodia at the cell periphery of SGC-7901 cells. (Left panel) The living cell image acquisition was performed at 25 °C with SGC-7901 cells transfected with GFP-RUFY3 and undergoing a scratch wound assay, and GFP vector was used as a control. A representative image was shown. The white boxed areas in the left images ( × 100; scale bars, 200 *μ*m) are magnified in the right images ( × 600; scale bars, 24 *μ*m). The red boxed area in the right images shows that the cells expressing GFP-RUFY3 can localize at the periphery in a scratch area. (Right panel) Histogram showed the relative percentage of cells with actin protrusion at the migrating edge. Data are the average of at least three independent experiments with similar results, in which ~100 cells were counted (**P<0.01, compared with GFP vector). Protein expression was confirmed by western blotting assays using GFP-tagged antibody when equal glyceraldehyde 3-phosphate dehydrogenase (GAPDH) was used as the endogenous reference protein. (**b** and **c**) RUFY3 colocalizes with F-actin at the cell periphery. SGC-7901 cells were transiently transfected with pEGFP-C1 or pEGFP-RUFY3. Rhodamine-conjugated phalloidin was used to detect F-actin. After 24 h transfection, cells were fixed and permeabilized. (**b**) Images were captured using a scanning confocal fluorescence microscope and one confocal section is shown in each image. Scale bars, 10 *μ*m. (**c**) Histogram showed the relative percentage of colocalization cells expressing GFP-RUFY3 with F-actin at the cell periphery. The data show mean±S.E.M. (**P<0.01, compared with GFP vector), in which ~40 transfected cells were observed. (**d**) Colocalization of GFP-RIPX and myosinIIb at the cell periphery is shown by confocal microscopy. SGC-7901 cells were transiently transfected with GFP vector or GFP-RIPX. Colocalization of myosinIIb (red) with GFP-RIPX is shown by yellow fluorescence. Scale bars, 10 *μ*m. (**e**) Colocalization of GFP-RIPX and integrin *β*5 at the cell periphery by plating cells on vitronectin is observed by confocal microscopy. SGC-7901 cells were transiently transfected with GFP vector or GFP-RIPX. Colocalization of integrin *β*5 (red) with GFP-RIPX is shown by yellow fluorescence. Scale bars, 20 *μ*m. (**f**) Colocalization of GFP-RIPX and integrin *α*3*β*1 at the cell periphery by plating cells on vitronectin is observed by confocal microscopy. Scale bars, 20 *μ*m

Previous studies suggested that RUFY3 was localized in growth cones in nerve cells.^17, 18^ Here, we detect the localization of RUFY3 in gastric cancer cell lines. The living cell image acquisition was performed at 25 °C with SGC-7901 cells transfected with GFP-RUFY3, and GFP vector was used as a control. Meanwhile, to further indicate the localization of GFP-RUFY3 at the migrating edge, we showed the localization of a cell undergoing a scratch wound assay and found that most of the SGC-7901 cells expressing GFP-RUFY3 formed the protrusion at the cell periphery by sequential scanning, and rarely detected in GFP-expressing cells (Figure 1a). We then analyzed whether RUFY3 colocalized with F-actin by confocal microscopy. As shown in Figure 1b, the GFP-RUFY3 was significantly colocalized with F-actin at the cell periphery in the majority of cells (Figure 1c). In addition, we measured the quantitation of colocalization with color scatterplots, and the analysis results showed that RUFY3 highly colocalized with F-actin at the cell periphery (Supplementary Figure S5a).

Although cell migration can be attributed to actin polymerization alone, other core migration components such as myosin, integrins and vinculin also have an important role in cell migration. We therefore decided to examine whether RUFY3 can colocalize with other core migration components. RUFY3 could be observed by the colocalization with myosinIIb (Figure 1d, Supplementary Figure S5b), integrin *β*5 (Figure 1e,Supplementary Figure S5c) and vinculin (Supplementary Figure S1) in some places of the cell periphery, but the colocalization of RUFY3 and integrin *α*3*β*1 at the cell periphery is not significant (Figure 1f). Taken together, these findings indicated that RUFY3 mainly localized in F-actin-enriched protrusion at the cell periphery.

### RUFY3 affects gastric cancer cell migration and invasion

**Figure 2:**
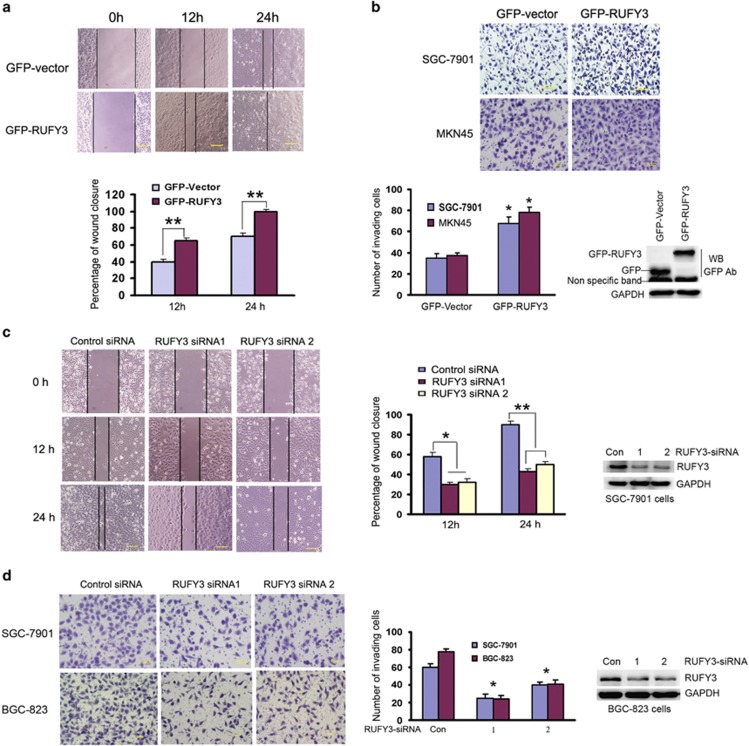
RUFY3 affects gastric cancer cell migration and invasion (**a** and **b**) Overexpression of RUFY3 promotes gastric cancer cell migration and invasion. (**a**) Cell motility was determined by assay measuring cell migration into the wound. (Upper panel) Representative photomicrographs of wound-healing results were taken under × 200 original magnification. Scale bars, 50 *μ*m. (Down panel) Histogram showed the percentage of wound closure. Data are the average of at least three independent experiments with similar results (*P <0.05, **P<0.01, compared with 0 h). (**b**) Cell invasion was determined by transwell assay with SGC-7901 and MKN45 cells transfected with pEGFP-vector or pEGFP-RUFY3. (Upper panel) Photographs represented the cells that traveled through the micropore membrane. Representative photomicrographs of transwell results were taken under × 200 original magnifications. Scale bars, 50 *μ*m. (Down panel) Number of invading cells is shown. The number of cells was counted in 16 independent symmetrical visual fields under the microscope ( × 400 original magnification) from three independent experiments (*P <0.05, **P<0.01, compared with control vector). Protein expression was confirmed by western blotting assays using GFP-tagged antibody when equal glyceraldehyde 3-phosphate dehydrogenase (GAPDH) was used as the endogenous reference protein. (**c** and **d**) Knockdown of RUFY3 inhibits gastric cancer cell migration and invasion. The different shRNAs (nos. 1 and 2) targeting RUFY3 were transfected into SGC-7901 and BGC-823 cells to perform the wound-healing and transwell invasion assays. (**c**) Photographs represented the cells migrating into the wounded area. (Left panel) Representative photomicrographs of wound-healing results were taken under × 200 original magnification. Scale bars, 50 *μ*m. (Middle panel) Histogram showed the relative migration distance of cells. Data are the average of at least three independent experiments with similar results (*P <0.05, **P<0.01, compared with 0 h). (Right panel) The efficacy of the RNAi of RUFY3 in SGC-7901 cells were determined by western blotting analysis using specific RUFY3 antibody, and the equal GAPDH was used as the endogenous reference protein.(**d**) Photographs represented the cells that traveled through the micropore membrane. (Left panel) Representative photomicrographs of transwell results were taken under × 200 original magnifications. Scale bars, 50 *μ*m. (Middle panel) Number of invading cells is shown. The number of cells was counted in 16 independent symmetrical visual fields under the microscope ( × 400 original magnifications) from three independent experiments (*P <0.05, **P<0.01, compared with control vector). (Right panel) The efficacy of the RNAi of RUFY3 in BGC-823 cells were determined by western blotting analysis using specific RUFY3 antibody, and the equal GAPDH was used as the endogenous reference protein

Actin protrusion at the cell periphery is central to the formation of invadopodia during tumor cell migration and invasion.^1, 2^ We next examined whether RUFY3 was able to affect the migration and invasion ability of the cells. Confluent human SGC-7901 cell transfected with pEGFP-C1 or pEGFP-RUFY3 were subjected to a wound-healing assay to monitor cell migration. The 70–80% transfection efficiency was determined by the expression of GFP by confocal microscopy (Supplementary Figure S2). An increase in wound-healing cell migration was clearly seen in cells expressing RUFY3 after 12 h compared with cells expressing GFP. Particularly, at 24 h, SGC-7901 cells expressing RUFY3 exhibited a significant increase compared with control cells expressing GFP (Figure 2a, left panel), and the relative migrating distance of cells was significantly longer (Figure 2a, right panel). Therefore, these data demonstrate that cells overexpressing RUFY3 have an increase in cell migration as measured in this classical wound-healing assay.

Cell migration is a critical step in tumor invasion and metastasis.^2^ Thus, we investigated whether the observed increase in cell migration in RUFY3-expressing cells was also associated with an increased invasion ability of these cells. A matrigel-coated Boyden chamber system was used to quantify the invasive ability of SGC-7901 cells (or MKN45 cells) expressing RUFY3. Remarkably, in the 24 h monitoring of cell invasion, the overexpression of RUFY3 leads to a significant increase in the ability of these cells to invade the reconstituted basement membrane (Matrigel) compared with GFP vector (Figure 2b, upper panel), and the relative number of invading cells was significantly increased (Figure 2b, down panel). However, there was no evidence to show GFP-RUFY3 promoted the proliferation and cell cycle progression compared with that seen in GFP vector (Supplementary Figure S3), suggesting that RUFY3 was not related to cell proliferation. These findings suggest that overexpression of RUFY3 may facilitate the migration and invasion of gastric cancer cells.

To demonstrate the importance of RUFY3-specific response in cell migration and invasion, endogenous RUFY3 in SGC-7901 cells and BGC-823 cells was knocked down by two different shRNAs (nos. 1 and 2) targeting RUFY3. The efficacy of RUFY3 shRNA was demonstrated by depletion of RUFY3. As expected, knockdown of RUFY3 inhibited gastric cancer cell migration and invasion compared with control siRNA cells (Figures 2c and d). Taken together, these above data indicate that RUFY3 can affect gastric cancer cell migration and invasion.

### PAK1 can associate with RUFY3 *in vitro* and *in vivo*

**Figure 3:**
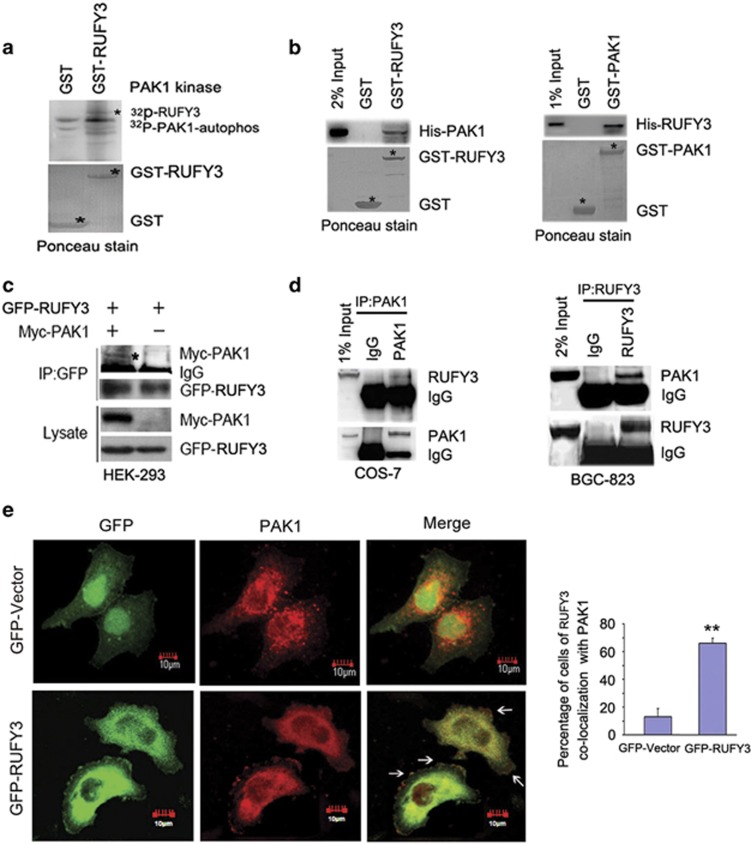
PAK1 can associate with RUFY3 *in vitro* and *in vivo.* (**a**) PAK1 phosphorylates RUFY3 *in vitro*. Glutathione *S*-transferase (GST) and GST-RUFY3 were used as PAK1 substrates in PAK1 kinase assay. (**b**) A specific interaction between RUFY3 and PAK1 was demonstrated by *in vitro* GST assay. Asterisks indicate GST, GST-RUFY3 and GST-PAK1 bands, and ponceau staining indicates the loading amounts. (Left panel) *In vitro*-translated His-PAK1 binds to purified GST-RUFY3. (Right panel) *In-vitro*-translated His-RUFY3 specifically interacts with GST-PAK1. (**c**) GFP-RUFY3 was co-precipitated with myc-PAK1. The cell lysate from HEK293 cells transfected with myc-PAK1 and GFP-RUFY3 were incubated with anti-GFP antibody. The immunoprecipitates were analyzed by western blot with anti-myc and anti-GFP antibodies. (**d**) Co-IP assays to identify endogenous PAK1 interacting with endogenous RUFY3 in COS-7 cells (left panel) and BGC-823 cells (right panel). *In vivo* anti-PAK1 antibody or anti-RUFY3 antibody immunoprecipitates endogenous RUFY3 or PAK1. Immunoblots were carried out as indicated. (**e**) Colocalization of PAK1 with GFP-RUFY3 at the cell periphery is shown by confocal microscopy. SGC-7901 cells were transiently transfected with GFP vector or GFP-RUFY3. (Left panel) Colocalization of PAK1 (red) with GFP-RUFY3 is shown by yellow fluorescence. Scale bars, 10 *μ*m. (Right panel) Histogram showed the relative percentage of colocalization cells expressing GFP-RUFY3 with PAK1 at the cell periphery. The data show mean±S.E.M. (**P<0.01, compared with GFP vector), in which ~40 transfected cells were observed

As the PAK1 kinase acts on its targets mainly through phosphorylation, we first investigated whether PAK1 might phosphorylate RUFY3. We found that PAK1 could phosphorylate GST-RUFY3 protein by *in vitro* kinase assays (Figure 3a), but the level of phosphorylation indicated is very weak. Therefore, based on our previous experimental data that RUFY3 was identified as a binding protein of PAK1 using yeast two-hybrid screen assays, we further examined whether PAK1 associated with RUFY3. GST pull-down assays demonstrated that *in vitro* translated PAK1 binds to GST-RUFY3 (Figure 3b, left panel). Conversely, RUFY3 protein specifically interacted with GST-PAK1 (Figure 3b, right panel). Human embryonic kidney 293 (HEK293) cells were transiently co-transfected with GFP-RUFY3 and myc-PAK1 and subjected to immunoprecipitation (IP) assay with GFP-tagged antibody. As shown in Figure 3c, GFP-RUFY3 was co-precipitated with myc-PAK1. We next performed co-IP and the results indicated that endogenous PAK1 specifically interacted with endogenous RUFY3 in COS-7 cells (Figure 3d, left panel) and BGC-823 cells (Figure 3d, right panel). It was also observed by confocal microscopy that GFP-RUFY3 colocalized with PAK1 (red) in actin protrusion at the cell periphery (yellow) (Figure 3e). The colocalization analysis further confirmed the results (Supplementary Figure S5d). In addition, to avoid the interference by GFP in RUFY3 localization, we constructed Flag-RUFY3 vector to perform the colocalization of Flag-RUFY3 and PAK1. It was observed my confocal microscopy that Flag-RUFY3 and PAK1 could colocalize at the cell periphery, which was consistent with the colocalization of GFP-RUFY3 and PAK (Supplementary Figure S5e).These data indicate that PAK1 can specifically interact with RUFY3 *in vitro* and *in vivo*.

### PAK1 positively regulates RUFY3 expression

**Figure 4:**
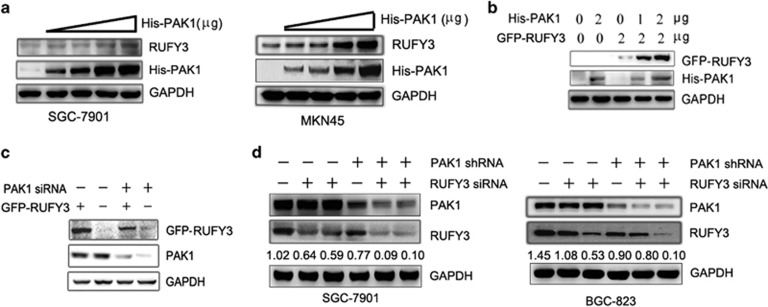
PAK1 positively regulates RUFY3 expression. (**a** and **b**) The protein level of RUFY3 was increased by the ectopic PAK1 expression level enhancing. (**a**) A dose-dependent increase of PAK1 plasmids were transfected into SGC-7901 cells (left panel) and MKN45 cells (right panel). Western blot assays were performed to detect the protein level of endogenous RUFY3. (**b**)The GFP-RUFY3 and the increasing amounts of PAK1 expression vector were transfected into SGC-7901 cells, after 24 h of transfection, the protein levels of His-PAK1 and GFP-RUFY3 were measured by western blot. (**c** and **d**) Inhibition of PAK1 expression can also decrease RUFY3 expression. (**c**) The SGC-7901 cells expressing GFP-RUFY3 were treated with PAK1-siRNA, and the protein levels of PAK1 and GFP-RUFY3 were measured by western blot. (**d**) The stable expressing PAK1-shRNA lentivirus SGC-7901 cells (left panel) and BGC-823 (right panel) cells were treated with two different shRNAs (nos. 1 and 2) targeting RUFY3, and the protein level of endogenous PAK1 and RUFY3 were measured by western blot

To further seek the correlation between PAK1 and RUFY3 and explore the underlying mechanism of RUFY3-mediated gastric cancer cell migration, the increasing amounts of PAK1 expression plasmids were transfected, and the western blot results indicated that the protein levels of endogenous and exogenous RUFY3 were increased by the ectopic PAK1 expression level increasing (Figures 4a and b), suggesting that overexpression of PAK1 promotes RUFY3 expression.

Then, to test the effect of PAK1 on RUFY3 expression by RNA interference, we treated the SGC-7901 cells expressing GFP-RUFY3 with PAK1-siRNA. As expected, compared with control siRNA, PAK1-siRNA decreased the GFP-RUFY3 expression (Figure 4c). Meanwhile we also treated the stable expressing PAK1-shRNA lentivirus SGC-7901 and BGC-823 cells with two different shRNAs (nos. 1 and 2) targeting RUFY3, and found that, compared with control-shRNA, PAK1-shRNA also significantly decreased endogenous RUFY3 expression (Figure 4d), suggesting that inhibition of PAK1 expression can decrease RUFY3 expression. Taken together, these results indicate that PAK1 can upregulate RUFY3 protein expression.

### PAK1 regulates RUFY3-mediated cell migration and invasion

**Figure 5:**
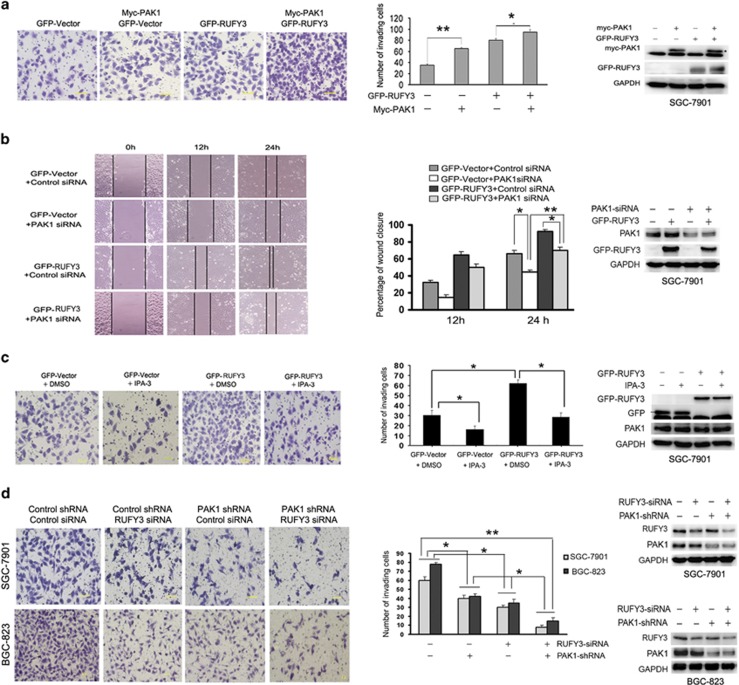
PAK1 regulates RUFY3-mediated cell migration and invasion. (**a**) Overexpression of PAK1 facilitates RUFY3-induced cell invasion. SGC-7901 cells were transfected with GFP-RUFY3 and myc-PAK1 or GFP vector and myc-PAK1, and were subjected to performing transwell invasion assay. Photographs represented the cells traveling through the micropore membrane. (Left panel) Representative photomicrographs of transwell results were taken under × 200 original magnifications. Scale bars, 50 *μ*m. (Middle panel) Number of invading cells is shown. The number of cells was counted in 16 independent symmetrical visual fields under the microscope ( × 400 original magnification) from three independent experiments (*P <0.05, **P<0.01, compared with control vector). (Right panel) The exogenous expression of RUFY3 and PAK1 were verified by western blot. (**b** and **c**) Inhibition of PAK1 attenuates RUFY3-mediated cell migration and invasion. (**b**)The SGC-7901 cells transfected with GFP-RUFY3 or GFP vector, which were along with PAK1-siRNA-treated (PAK1 siRNA) or control siRNA-treated (Control siRNA), were used to perform the wound-healing assay. (Left panel) Photographs represented the cells migrating into the wounded area. Scale bars: 50.0 *μ*m. (Middle panel) Histogram showed the percentage of wound closure. Data are the average of at least three independent experiments with similar results (*P <0.05, **P<0.01, compared with 0 h). (Right panel) The efficacy of PAK1-shRNA was determined by western blot. (**c**) SGC-7901 cells transfected with GFP-RUFY3 were treated with or without IPA-3 (5 *μ*M) for 24 h, Me2SO (DMSO) as a control, and used to perform the transwell invasion assay. Photographs represented the cells traveling through the micropore membrane. (Left panel) Representative photomicrographs of transwell results were taken under × 200 original magnifications. Scale bars, 50 *μ*m. (Middle panel) Number of invading cells is shown. The number of cells was counted in 16 independent symmetrical visual fields under the microscope ( × 400 original magnification) from three independent experiments (*P <0.05, **P<0.01, compared with control vector). (Right panel) The exogenous expression of RUFY3 was demonstrated by western blotting when cells were treated with or without IPA-3. (**d**) Knockdown of both PAK1 and RUFY3 can significantly inhibit cell invasion. The RUFY3-shRNA1 was transfected into stable expressing PAK1-shRNA lentivirus SGC-7901 cells and BGC-823 cells to perform the transwell invasion assay. Photographs represented the cells traveling through the micropore membrane. (Left panel) Representative photomicrographs of transwell results were taken under × 200 original magnifications. Scale bars, 50 *μ*m. (Middle panel) Number of invading cells is shown. The number of cells was counted in 16 independent symmetrical visual fields under the microscope ( × 400 original magnifications) from three independent experiments (*P <0.05, **P<0.01, compared with control vector). (Right panel) The efficacys of PAK1-shRNA and RUFY3-siRNA were determined by western blot

Next, we decided to examine whether PAK1 can also influence the RUFY3-mediated cell migration and invasion. As expected, a significant increase in wound- healing cell migration was clearly seen in SGC-7901 cells expressing RUFY3 and PAK1 compared with cells expressing RUFY3 alone (Supplementary Figure S4a, left panel), and the relative migrating distance of cells was significantly longer (Supplementary Figure S4a, right panel). In addition, a matrigel-coated Boyden chamber system was used to quantify the invasive ability of SGC-7901 cells expressing RUFY3 and PAK1. Remarkably, at the 24 h, the effect of promoting SGC-790 cell invasion induced by RUFY3 can be more significantly facilitated by overexpressing PAK1 compared with cells expressing RUFY3 alone (Figure 5a, left panel), and the relative number of invading cells was significantly increased (Figure 5a, right panel), indicating that PAK1 as an important role in RUFY3-mediated facilitating gastric cancer cell migration and invasion. These results suggest that overexpressing PAK1 facilitates RUFY3-mediated migration and invasion of gastric cancer cells.

Besides, we sought to test the role of PAK1 in RUFY3-induced SGC-7901 cell migration and invasion by RNA interference and using PAK1 inhibitor IPA-3. Nowadays many small-molecule inhibitors were reported targeting PAKs, especially PAK1.^28, 29^ It was also reported that IPA-3, a highly specific and potent non-ATP-competitive inhibitor, targeted the autoregulatory domain of PAK1.^28, 30^ Next, we therefore investigated the effects of PAK1 on the RUFY3-induced SGC-7901 cell migration using RNA interference and PAK1 inhibitor IPA-3. The SGC-7901 cells expressing GFP-RUFY3, which were treated with PAK1-siRNA, exhibited an inhibition capacity in cell migration (Figure 5b) and invasion (Supplementary Figure S4b) compared with cells treated with negative control siRNA. In addition, these invading assays indicated a dose-dependent increase of invading cells in RUFY3-overexpressing sample with low levels of IPA-3 (Supplementary Figure S4c), and the expressing GFP-RUFY3 cells treated with IPA-3 showed consistent results with PAK1-siRNA (Figure 5c and Supplementary Figure S4d). All together, the results indicate that inhibition of PAK1 attenuates RUFY3-induced cell migration and invasion.

In addition, to detect the effect of PAK1 on RUFY3-siRNA cell migration and invasion when PAK1 was blocked, we treated the stable expressing PAK1-shRNA lentivirus SGC-7901 cells with two different shRNAs (nos. 1 and 2) targeting RUFY3, and found that the inhibitory effect of cell migration (Supplementary Figure S4e) and invasion (Figure 5d, upper panel) is significantly enhanced by knockdown of both PAK1 and RUFY3, which were also shown in BGC-823 cells (Figure 5d, down panel), suggesting that blocking the expression of PAK1 and RUFY3 can significantly inhibit gastric cancer cell migration and invasion.

### The positive correlation between the expression of RUFY3 and PAK1 in gastric cancer cells and clinical gastric cancer tissue samples

**Figure 6:**
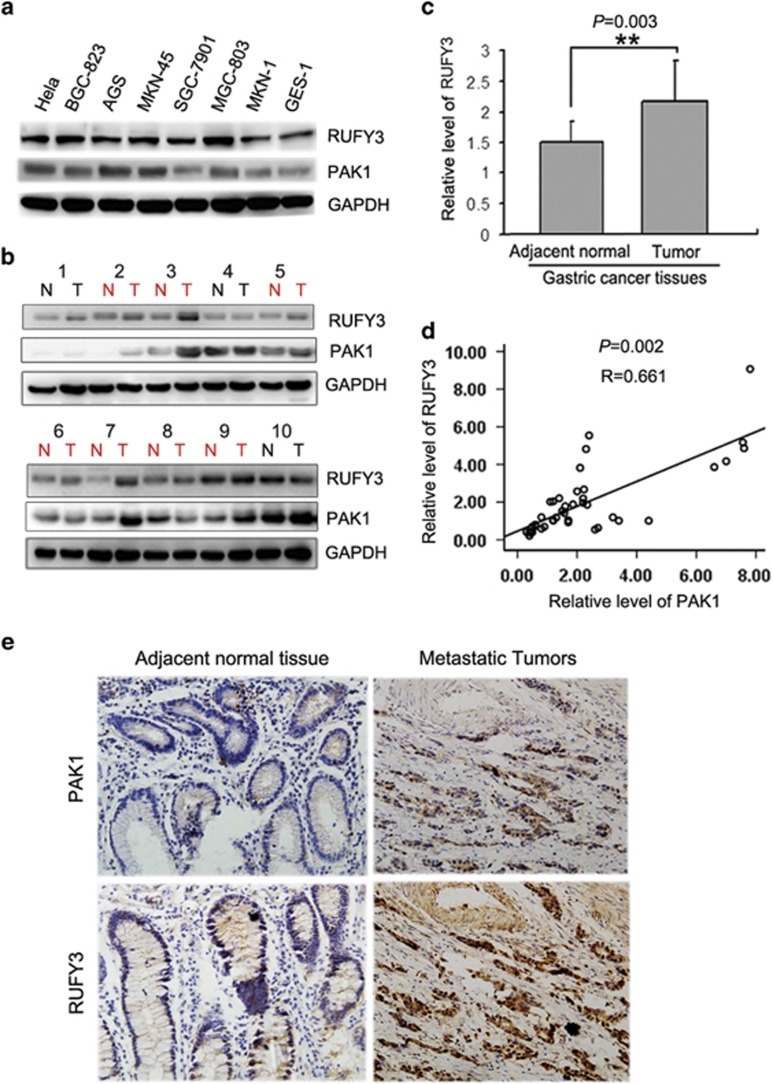
The positive correlation between RUFY3 and PAK1 in gastric cancer cells and clinical gastric cancer tissue samples. (**a**) The protein levels of RUFY3 in gastric cancer cell lines (BGC-823, MKN45, AGS MGC-803, SGC-7901, BGC-823 and MKN1) relative to the normal gastric epithelial cell line (GES-1) were analyzed by western blot. (**b**) The protein level of RUFY3 is positively correlated with PAK1 in gastric cancers and matched adjacent normal gastric tissue samples. Total protein from gastric cancer samples was extracted, and the protein levels of RUFY3 and PAK1 were measured by western blot. (**c**) Histogram showed the relative level of RUFY3 in gastric cancer tissues. Among 40 patients with gastric cancer, 28 of 40 (70%) samples revealed >50% increase in the RUFY3 level relative to their matched non-tumor adjacent tissues (*P*=0.003). (**d**) The relative protein level of RUFY3 was plotted against that of PAK1 in gastric cancers tissue samples with Spearman's correlation statistical analysis from (**b**). Spearman's correlation coefficient is 0.661 (*P*=0.002). (**e**) Immunohistochemical analyses of RUFY3 and PAK1 expression in adjacent normal gastric tissues and metastatic gastric cancer. Scale bar, 50 *μ*m

To examine the role of RUFY3 and its relationship with PAK1 in gastric cancer, we first investigated the protein levels of RUFY3 and PAK1 in gastric cancer cell lines (BGC-823, MKN45, AGS, MGC-803, SGC-7901 and MKN1) relative to the normal gastric epithelial cell line (GES-1) by western blot. As shown in Figure 6a, compared with GES-1, RUFY3 and PAK1 were highly expressed in BGC-823, MKN45, AGS and MGC-803 cells. In addition, we measured the protein levels of RUFY3 and PAK1 in 40 pairs of gastric cancer tissue samples with invasive carcinoma at pathologic TNM III and TNM IV stages through western blot. Among 40 patients with gastric cancer, 28 of 40 (70%) samples revealed >50% increase in the RUFY3 level relative to their matched non-tumor adjacent tissues (Figures 6b and c, *P*=0.003 and Table 1), and PAK1 had high expression as well in most of the gastric cancer tissues, which was consistent with the previous reports on PAK1 expression in cancer cells and gastric cancer samples.^18, 20^ Moreover, RUFY3 levels in gastric cancer sample were also analyzed and plotted against the level of PAK1. The 10 representative samples of gastric cancer showed a positive correlation expression between PAK1 and RUFY3 (Figure 6b). After quantifying the protein fragments, an obvious positive correlation was observed between RUFY3 and PAK1 expression in tumor tissue samples (*P*=0.002; Table 2), and the Spearman's correlation coefficient was perfect (Figures 6d, *R*=0.661).

We also analyzed the levels of RUFY3 and PAK1 by immunohistochemistry in 40 gastric cancers specimen including metastatic tumors. The results also showed that high levels of PAK1 and RUFY3 appear to be associated with the progression and metastasis of human gastric cancer, which indicated that the expression level of RUFY3 by immunohistochemistry are correlated with PAK1, consistent with western blot results. Representative samples were shown in Figure 6e.

## Discussion

Cell migration is a crucial event in cancer metastasis.^1, 2^ It is generally known that the redistribution of actin fibers and the formation of pseudopodia are important events in cell migration,^3^ other core migration components such as myosin and integrins also play an important role in cell migration. Different types of integrins have different roles in cell adhesion and migration. Therefore, in our study, in addition to F-actin, we also observe the colocalization of RUFY3 with myosinIIb and integrin *β*5. Based on these findings, we suggest that RUFY3 may participate in cell migration.

Our data show that overexpressing PAK1 consolidated overexpressing RUFY3-induced gastric cancer cell migration and invasion, whereas the cells overexpressing GFP-RUFY3 treated with PAK1-siRNA exhibited an inhibition capacity in cell migration and invasion. In addition, we found that the inhibitory effect of cell migration and invasion is significantly enhanced by knockdown of both PAK1 and RUFY3 compared with knockdown of RUFY3 alone or PAK1 alone. Therefore, our results indicated that PAK1 regulated RUFY3-mediated gastric cancer cell migration and invasion.

Up to date, the function of RUFY3 has been rarely reported, especially in cancer cells. The RUFY family contains RUFY1/Rabip4,^31^ RUFY2,^16^ RUFY3/SINGAR1/RUFY3 and RUFY4; they share an N-terminal RUN domain and one or two coiled-coil domains in their C-terminal end.^8^ Besides, RUFY3, RUFY1, RUFY2 and RUFY4 contain an additional FYVE (Fab1, YOTB/ZK632.12, Vac1 and EEA1) domain in their C terminal.^32^ Most proteins containing an FYVE domain have been found to localize in early endosomes and to have a role in membrane trafficking, cytoskeleton remodeling and signal transduction.^16, 32, 33^ Therefore, we think, it will be further studied whether RUFY3 lacking an FYVE domain can localize in endosome through binding Rab protein to participate in Rab-mediated membrane trafficking, although it was considered be likely to interact with Rab33A.^8^ Here, we demonstrated that RUFY3/RUFY3 could directly interact with PAK1 and colocalized in F-actin-enriched invadopodia at the cell periphery to promote cancer cell migration and invasion. Meanwhile, it would be interesting for us to investigate whether the interaction between RUFY3 and PAK1 requires Rab-binding activity to participate in Rab-mediated membrane trafficking in the future.

In previous study, the relationship between RUFY3 and PAK1 pathways in cancer has not been described. This is the first study of the relationship between the RUFY3 and PAK1 pathway. Mounting evidence has confirmed that PAK1 signaling pathway has a central role in cancer cell migration and invasion.^22, 23, 34^ Overexpression of PAK1 has been correlated with human cancer invasiveness and tumor grade.^21, 26^ Importantly, in this study, we found that relative protein expression of RUFY3 was high in clinical gastric cancer samples with invasive carcinoma at pathologic TNM III and TNM IV stages compared with their matched non-tumor adjacent tissues. We also found that the protein expressions of RUFY3 and PAK1 positively correlate with human clinical gastric cancer samples. Using specific PAK1-shRNA interference and inhibitor IPA-3, there was a reduction in RUFY3-induced gastric cancer cell migration and invasion. Here, we found that PAK1 and RUFY3 concordantly regulated cell migration and invasion by evaluating the effects of RUFY3 and PAK1, alone or in combination. These findings suggest that RUFY3 associates with an invasive phenotype of clinical gastric cancer by interacting with PAK1, which shows a novel function of RUFY3.

Therefore, based on these results, we draw a conclusion that PAK1 regulates RUFY3-mediated gastric cancer cell migration and invasion, suggesting that blocking PAK1-RUFY3 pathway might be a potential therapeutic strategy for metastasis of gastric cancer. These findings also will broaden the understanding of PAK1 in tumor cell signaling pathway.

## Materials and Methods

### Tissue samples

Samples of human gastric cancer tissues and paired-adjacent non-tumor gastric tissues that were 5 cm away from the tumors were obtained from 40 patients who underwent radical resection at the First Hospital of China Medical University (Shenyang, China). Fresh samples were snap frozen in liquid nitrogen immediately after resection and stored at −80 °C. The samples were obtained with patients' informed consent and were histologically confirmed by staining with hematoxylin–eosin. The histological grade of cancers was assessed according to the criteria set by the World Health Organization. All research involving human participants have been approved by the First Hospital of China Medical University ethics committees.

### Cell cultures, transfection and RNA interference

Human gastric cancer cell lines BGC-823, SGC-7901, MKN-45, HEK293 and COS-7, were cultured in DMEM (Invitrogen, Carlsbad, CA, USA) supplemented with 10% fetal calf serum (Invitrogen) at 37 °C in an incubator with humidified atmosphere of 5% CO_2_ and 95% air. *In vitro* transfections were achieved with Lipofectamine 2000 reagent (Invitrogen) following the manufacturer's protocols. SGC-7901 cells were incubated with IPA-3 (5 *μ*M) for 24 h (Sigma, St. Louis, MO, USA). The chemically synthesized PAK1-siRNA and negative control siRNA were purchased from Cell Signaling (Danvers, MA, USA). PAK1-shRNA lentivirus was purchased from Shanghai GeneChem Company (Shanghai, China). Commercial lentivirus was used to infect BGC-823 cells in a 12-well plate with 3 mg/ml polybrene. Infected BGC-823 cells were indentified by western blot. The target siRNA sequences for human RUFY3 were: 5′-TCTCAAGCATGAACTTGCCTTTAAG-3′ (no. 1) and 5′-GACTAATCAGATGGCTGCTACCATT-3′ (no. 2). Control siRNA sequences were: 5′-UUCUCCGAACGUGUCACGUTT-3′. PAK1-shRNA lentivirus BGC-823 and SGC7901 cells were transfected with either RUFY3 siRNAs or non-silencing siRNA (as a negative control). All the siRNAs were synthesized by GenePharma (Shanghai, China). Transfection was performed with Lipofectamine 2000 reagent (Invitrogen) following the manufacturer's protocol. A final concentration of 100 nM of siRNA and their respective negative controls were used for each transfection in wound- healing and cell invasion assays. Transfection efficiency was monitored by western blot.

### Expression vectors

The human RUFY3 cDNA clone was obtained from the National Institutes of Health Mammalian (Bethesda, MD, USA) Gene Collection (NIH-MGC; http://mgc.nci.nih.gov). The full-length RUFY3 coding sequence was ligated into pGEX-4T-2 (Promega, Madison, WI, USA) and pEGFP-C1 (Clontech, Mountain View, CA, USA) using the *Xho*I and *BamH* I sites to construct expression vectors of GST-RUFY3 and GFP-RUFY3. The specific PCR primers are shown as follows: 5′-GGTCCCACTCGAGTCATCATGTCTGCT-3′ and 5′-CGGACTTGCGACGGATCCAACTTA-3′, and the construct was verified by sequencing. PAK1 plasmids were a gift from J Chernoff (Fox Chase Cancer Center, Philadelphia, PA, USA).

### Immunofluorescence, time-lapse image acquisition and confocal microscopy analysis

SGC7901 cells transfected with GFP vector or GFP-RUFY3 grown on glass coverslips were fixed in methanol at room temperature for 15 min, and then blocked with normal goat serum for 1 h. The cells were incubated with rhodamine-conjugated phalloidin (Sigma) to detect F-actin for 1 h at room temperature; rabbit anti-integrin *α*3*β*1 (1 : 50; Bioss Inc.), mouse anti-integrin *α*3*β*1 (1 : 100; Abcam); rabbit anti-vinculin (1 : 100; Santa Cruz, Santa Cruz, CA, USA), rabbit anti-myosinIIb, integrin *β*5 and Flag-tagged (1 : 100; Shanghai, Kangcheng, Shanghai, China), PAK1 (1 : 50; Cell Signaling) antibody were used overnight at 4 °C and rabbit anti-goat Alexa-546 secondary antibody (1 : 100; Molecular Probes) was used for 1 h at room temperature, and washed three times in PBT (PBS with 1‰ Triton X-100). The DNA dye DAPI was used to costain the DNA (blue). Confocal scanning analysis was performed by using a Leika laser confocal scanning microscope (Solms, Germany) in accordance with established methods, using sequential laser excitation to minimize the possibility of fluorescent emission bleed-through.

The living cell image acquisition was performed at 25 °C. Single scans and serial images were acquired using a confocal laser scanning microscope (UltraVIEWVoX; Perkin-Elmer, Madison, WI, USA). Cells were observed with a x60 objective lens. EGFP fluorescence was excited with the 488-nm line of an argon laser. Emission light was filtered through a 505-nm long-pass filter. For data analysis, the entire cell was imaged under non-saturating conditions. Time-lapse image capturing and data evaluation were performed using the image analysis software (Volocity demo; Perkin-Elmer).

### Western blot and IP assay

To determine the expression of protein, whole-cell extracts were prepared from 1 × 10^6^ cells in RIPA lysis buffer (50 mM Tris-HCl, pH 7.4, 150 mM NaCl, 1% Nonidet P-40, 0.25% Na-deoxycholate, 1 mM EDTA and protease inhibitor cocktail). Equal amounts of denatured protein were separated by SDS-PAGE and transferred to a PVDF membrane (Millipore, Billerica, MA, USA). The membrane was blocked with 5% nonfat dry milk in TBS-T (20 mM Tris, pH 7.4, 137 mM NaCl, 0.05% Tween-20) for 3 h at room temperature, and the proteins were probed with specific antibodies: GFP and His (GenScript Corporation, Nanjing, China), Flag (Shanghai Kangcheng), PAK1, integrin *β*5 and myosinIIb (Cell Signaling), RUFY3 and vinculin (Santa Cruz), integrin *α*3*β*1 (Bioss Inc. and Abcam). All PVDF membranes were detected by chemiluminescence (ECL; Thermo Fisher Scientific, Pierce Technology, Pittsburgh, PA, USA). To assure equal loading, membranes were stripped and reprobed with antibody against GAPDH (Shanghai Kangchen). For IP, cells were washed with ice-cold PBS two times before being lysed in IP lysis buffer (25 mM Tris, pH 7.4, 150 mM NaCl, 1% Nonidet P-40, 1 mM EDTA) supplemented with proteinase and phosphatase inhibitors (2 mM dithiothreitol, 1 mM phenylmethylsulfonyl fluoride, 10 *μ*g/ml leupeptin, 10 *μ*g/ml aprotinin, 20 mM glycerophosphate, 1 mM Na_3_VO_4_). Then, the supernatants with equal amounts of protein were subjected to IP using GFP-tagged antibody, RUFY3 or PAK1 antibodies and the protein A-Sepharose 4B beads (GE Healthcare Bio-Science, Piscataway, NJ, USA). The precipitated proteins were denatured in 2 × SDS loading buffer, separated by SDS-PAGE, transferred to PVDF membrane and analyzed by western blot.

### GST pull-down and PAK1 kinase assays

GST and GST-fusion RUFY3 proteins were purified with glutathione-conjugated Sepharose beads (Amersham Biosciences, Piscataway, NJ, USA) *in vitro*. The amounts of GST-fusion proteins were stained by Ponceau. For *in vitro* GST pull-down assay, *in vitro* transcription and translation of the PAK1 and RUFY3 proteins were performed by using the TNT-coupled transcription–translation system (Promega). Using a T7-TNT Kit, we translated 1 *μ*g of pcDNA-3.1 vector in the presence of ^35^S-labeled methionine in a reaction volume of 50 *μ*l. An aliquot of 10 *μ*l was used for each GST pull-down assay. Translation protein size was verified by subjecting 1 *μ*l reaction mixture to SDS-PAGE and autoradiography. Ponceau stain indicated the loading amounts of the GST-fusion proteins. The bound proteins were then visualized by western blot using anti-His antibody. PAK1 kinase assay used in this study have been described previously in detail.^19^

### Wound-healing assays

SGC-7901 cells were grown to 75% confluence in 35-mm dishes at the time of transfection. At 24 h after transfection, cell cultures reach 95% confluence. To determine the area of wound, we gently and slowly scratched the monolayer with a new 200 *μ*l pipette tip across the center of the well. Choose five gap distance equals to the outer diameter of the end of the tip. After scratching, gently wash the well two times with a medium to remove the detached cells, and the well was replenished with a fresh medium. Take photos for the stained monolayer on a microscope. Set same configurations of the microscope when taking pictures for different views of the stained monolayer. The progress of cells moving into the wound area was photographed at 0, 12 and 24 h using Olympus inverted microscope (Tokyo, Japan) at x20 magnification. The gap distance can be quantitatively evaluated, and each experimental group should be repeated three times. The relative migration distance of cells was measured by the initial distance minus the non-filled in area. The data show mean±S.E.M.

### Cell invasion assay

Matrigel cell invasion assays were performed by using Matrigel Invasion Boyden Chamber (BD Biosciences) according to the manufacturer's instructions. Precoated filters (6.5 mm in diameter, 8-*μ*m pore size, Matrigel 100 *μ*g/cm^2^) were rehydrated with 100 *μ*l medium. Then, 1 × 10^5^ cells in 100 *μ*l serum-free DMEM supplemented with 0.1% bovine serum albumin were placed in the upper part of each chamber, whereas the lower compartments were filled with 600 *μ*l DMEM containing 10% serum, which was determined by a previously described method.^17^

### Immunohistochemistry

Paraffin-embedded gastric tumor tissues were obtained from the First Hospital of China Medical University. Five-micrometer-thick consecutive sections were cut and mounted on glass slides. The glass slides were deparaffinized and then rehydrated before antigen retrieval, by blocking endogenous peroxidases. The sections were then washed three times in 0.01 mol/l PBS for 5 min each and blocked for 1 h in 5% normal goat serum. The sections were exposed to RUFY3 (1 : 100) and anti-PAK1 (1 : 200) 4 °C overnight. After brief washes in 0.01 mol/l PBS, sections were exposed for 2 h to 0.01 mol/l PBS containing horseradish peroxidase-conjugated goat anti-rabbit immunoglobulin G (1 : 200), followed by development with 0.003% H_2_O_2_ and 0.03% 3, 3′-diaminobenzidine in 0.05 mol/l Tris-HCl.

All of the immunostained sections were reviewed by two authors who had no knowledge of the patients' clinical status. Five areas selected at random were scored. All sections were scored in a semiquantitative manner according to a previously described method, which reflects both the intensity and percentage of cells staining at each intensity.^35^ Intensity was classified as 0 (no staining), +1 (weak staining), +2 (distinct staining) or +3 (very strong staining). A value designated as the ‘HSCORE' was obtained for each slide by using the following algorithm: HSCORE=∑ (*I* × PC), where *I* and PC represent the staining intensity and the percentage of cells that stain at each intensity, respectively. And the corresponding HSCOREs were calculated separately.

### Statistical analysis

Quantitative data are presented as mean values±S.E.M. from ≥3 independent repetitions. A *P*-value <0.05 was considered as statistically significant.

## Figures and Tables

**Table 1:** Expressions of RUFY3 in gastric cancer tissues and adjacent noncancerous tissues

**Characteristic**	**RUFY3**		
	**Case (*n*)**	**Mean±S.D.**	**P-value**
Adjacent noncancerous tissue	20	1.43±0.31	0.003^a^
Cancer tissues	20	2.06±0.60	

a Indicated statistical significance (*P*<0.05).

**Table 2:** Correlation between the expression of RUFY3 and PAK1 protein in 40 gastric cancer samples

	**RUFY3**^a^		***n***	***P*****-value**	**Spearman's correlation**	
	**Higher**	**Lower**			***R***-**value**	***P*****-value**
*PAK1* b						
Higher	26	2	28	0.0001^c^	0.661	0.002^d^
Lower	2	10	12			

a Number of cancers with reduced or increased levels of RUFY3 relative to non-tumor adjacent tissues.

b Number of cancers with reduced or increased levels of PAK1 protein relative to non-tumor adjacent tissues.

c Indicated statistical significance (*P*<0.05).

d Indicated statistical significance (*P*<0.01, two-tailed)

## Abbreviations

RUFY3: RUN and FYVE domain containing 3
RIPX: Rap2 interacting protein X
PAK1: p21-activated kinase-1
RUN: RPIP8, Unc-14 and NESCA domain
Singar1: single axon-related1
IP: immunoprecipitation
