# Supplementary material for: PAK1 regulates RUFY3-mediated gastric cancer cell migration and invasion
Source: Cell Death Dis. 2015 Mar 12;6(3):e1682–. doi: 10.1038/cddis.2015.50 (PMC4385928; doi:10.1038/cddis.2015.50)
Supplement: Supplementary Information [file cddis201550x1.doc]

**Supplement** **figure legends**

**Figure S1** Co-localization of vinculin and GFP-RUFY3 at the cell periphery is shown by confocal microscopy. SGC-7901 cells were transiently transfected with GFP vector or GFP-RUFY3. Co-localization of vinculin (red) with GFP-RUFY3 is shown by yellow fluorescence. Scale bars: 10μm.

**Figure S2** The transfection efficiency was determined with the expression of GFP by confocal microscopy both confocal microscope and phase contrase microscope with the different magnification (4×, 10× and 20×) .

**Figure S3** The effect of RUFY3 expression on cell proliferation of the gastric cancer cells

**(a)** The effect of RUFY3 expression on growth rate of the gastric cancer cells was measured by MTT proliferation assays. 2×10^3^ cells were seeded into 96-well culture plates with SGC-7901 cells transfected with GFP vector or GFP-RUFY3. After 24 hours of transfection, the cells were treated with MTT for 4 hours, and absorbance was measured at 12, 24, 36 and 48 h, respectively. Results represent the mean ± SD of 3 experiments done in triplicate. Optical density (OD) was determined using a spectrophotometer (Bio-Rad) at a wavelength of 490 nm. **(b and c)** The effect of RUFY3 expression on cell cycle progression of the gastric cancer cells was measured by flow cytometry. **(b)**SGC-7901 cells transfected with GFP vector or GFP-RUFY3 were stained with propidium iodide and cell-cycle distribution was analyzed by flow cytometry. Results represent the mean ± SD of 3 experiments done in triplicate. **(c)** A representative image was showed at least three independent experiments with similar results.

**Figure S****4** **PAK1 regulates RUFY3-mediated cell migration and invasion**

**(a)** Overexpression of PAK1 facilitates RUFY3-induced cell migration**.** SGC-7901 cells were transfected with GFP-RUFY3 and myc-PAK1 or GFP vector and myc-PAK1, and were subjected to performing the wound healing assay. Cell motility was determined by wound-healing assay measuring cell migration into the wound. *(left panel)* Photographs represented the cells migrated into the wounded area. Scale bars: 50.0μm. *(right panel)*Histogram shown percentage of wound closure. Data are the average of at least three independent experiments with similar result (*P <0.05, **P<0.01, compared with 0h).**(b-d)** Inhibition of PAK1 attenuates RUFY3-mediated cell migration and invasion.**(b)**The SGC-7901 cells transfected with GFP-RUFY3 or GFP Vector, which were along with PAK1 siRNA (PAK1-siRNA) or control siRNA (Control-siRNA), were used to perform the transwell invasion assay. Photographs represented the cells travelled through the micropore membrane. (*upper panel*)Representative photomicrographs of transwell results were taken under ×200 original magnification. Scale bars, 50.00 μm.(*down panel*) Number of invading cells is shown. The number of cells was counted in 16 independent symmetrical visual fields under the microscope (×400 original magnification) from three independent experiments (*P <0.05, **P<0.01, compared with control vector). **(c)**The transwell invading assays observed a dose-dependent increase of invading cells in RUFY3 overexpressing sample with low levels of IPA3. Photographs represented the cells travelled through the micropore membrane. (*upper panel*)Representative photomicrographs of transwell results were taken under ×200 original magnification. Scale bars, 50.00 μm.(*middle panel*) Number of invading cells is shown. The number of cells was counted in 16 independent symmetrical visual fields under the microscope (×400 original magnification) from three independent experiments (*P <0.05, **P<0.01, compared without IPA3). (*down panel*) The exogenous expression of RUFY3 was demonstrated by Western blotting when cells were treated with low levels of IPA3. GFP vector as a control. **(d)** The SGC-7901 cells transfected with GFP-RUFY3 were treated with or without IPA-3 (5uM) for 24 hours, Me2SO (DMSO) as a control, and used to perform the wound healing assay.Photographs represented the cells migrated into the wounded area. (*upper panel*)Representative photomicrographs of wound-healing results were taken under ×200 original magnification. Scale bars, 50.00 μm. (*down panel*) Histogram shown percentage of wound closure. Data are the average of at least three independent experiments with similar results (*P <0.05, **P<0.01, compared with 0h). **(e)** Knockdown of both PAK1 and RUFY3 synergistically inhibit cell migration. The RUFY3 shRNA#1 was transfected into stable expressing PAK1-shRNA lentivirus BGC-823 cells to perform the wound healing assay. Photographs represented the cells migrated into the wounded area. (*upper panel*)Representative photomicrographs of wound-healing results were taken under ×200 original magnification. Scale bars, 50.00 μm. (*down panel*)Histogram shown percentage of wound closure. Data are the average of at least three independent experiments with similar results (*P <0.05, **P<0.01, compared with 0h).

**Figure S5** The measurement for co-localization with *Image J analysis*. The intensity correlation analysis (ICA) plots were shown, one plot for the green (RUFY3) channel, one plot for the red (F-actin, myosinIIb, integrinβ5 or PAK1).The axes on the plots are the PDM values on the x-axis and the red or green intensity on the y-axis. The PDM value is the *P*roduct of the *D*ifferences from the *M*ean, i.e. for each pixel: PDM = (red intensity- mean red intensity)×(green intensity – mean green intensity).Colour Scatter plots of red intensities vs green intensities were shown. R (Mander’s Overlap coefficient) ranges between 1 and zero with 1 being high-colocalisation, zero being low. The intensity correlation quotient (ICQ) values are distributed between -0.5 and +0.5 by subtracting 0.5 from this ratio,with Random (or mixed) staining ICQ=0; dependent staining 0＜ICQ ≤+0.5, and for segregated staining 0＞ICQ≥-0.5.

(a-d)These co-localization results of GFP-RUFY3 and F-actin (Figure S5a, R=0.923, ICQ=0.393), myosin IIb (Figure S5b, R=0.781, ICQ=0.322), integrin β5 (Figure S5c, R=0.811, ICQ=0.237) and PAK1 (Figure S5d, R=0.93, ICQ=0.37) at cell periphery were shown by *Image J analysis*. These results indicated that RUFY3 could highly colocalize with these protein at cell periphery. (e) The co-localization of Flag-RUFY3 and PAK1 could be observed by confocal microscopy at cell periphery. SGC-7901 cells were transiently transfected with Flag-RUFY3 or Flag control vector. Co-localization of PAK1 (red) with Flag-RUFY3 is shown by yellow fluorescence. Scale bars: 20μm. Protein expression was confirmed by western blotting assays using Flag-tagged antibody, the equal GAPDH was used as the endogenous reference protein.
