# Supplementary figures and images for: PAK1 regulates RUFY3-mediated gastric cancer cell migration and invasion
Source: Cell Death Dis. 2015 Mar 12;6(3):e1682–. doi: 10.1038/cddis.2015.50 (PMC4385928; doi:10.1038/cddis.2015.50)

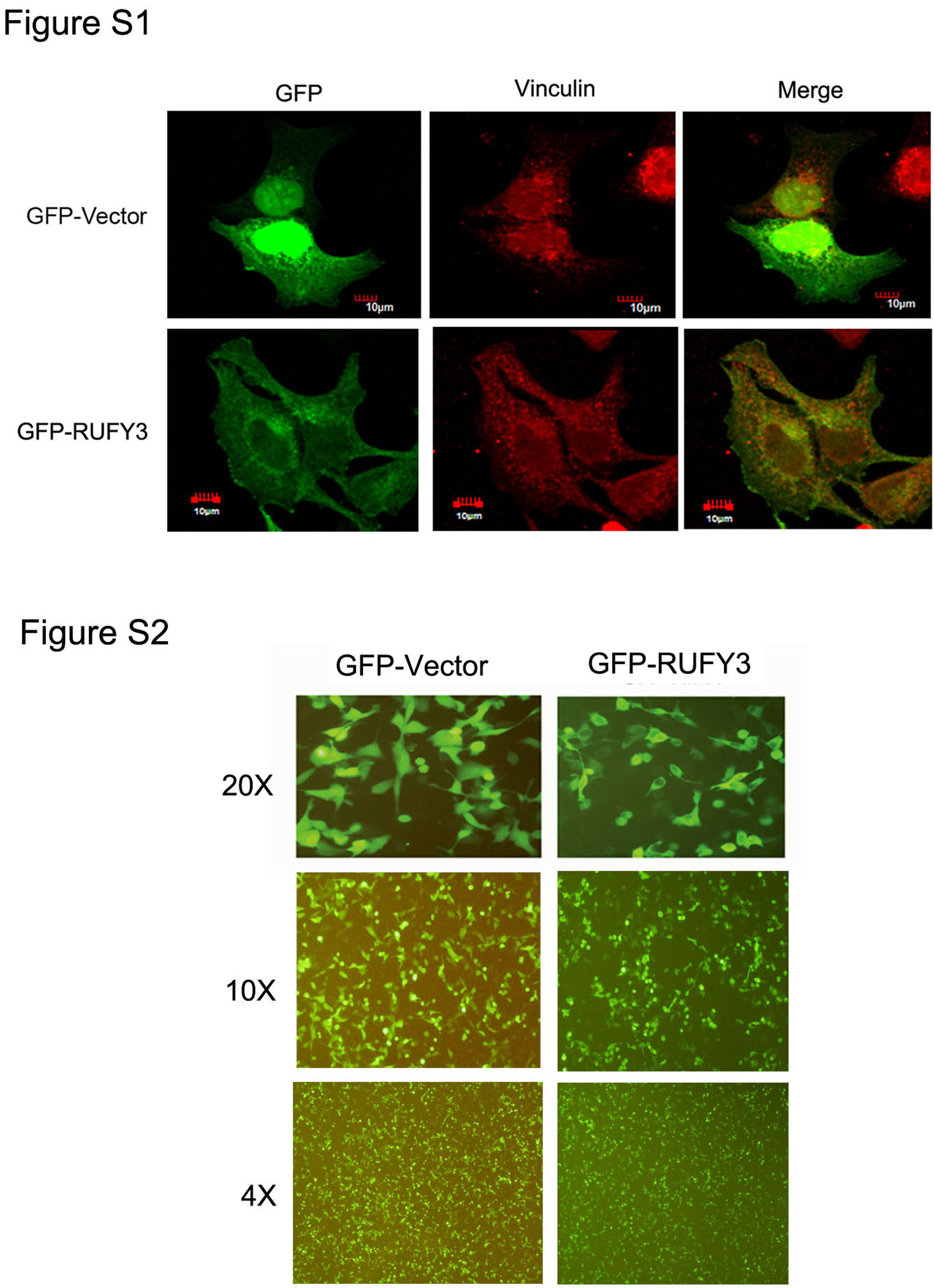

Supplement: Supplementary Figure 1 and 2 [file cddis201550x2.tif]

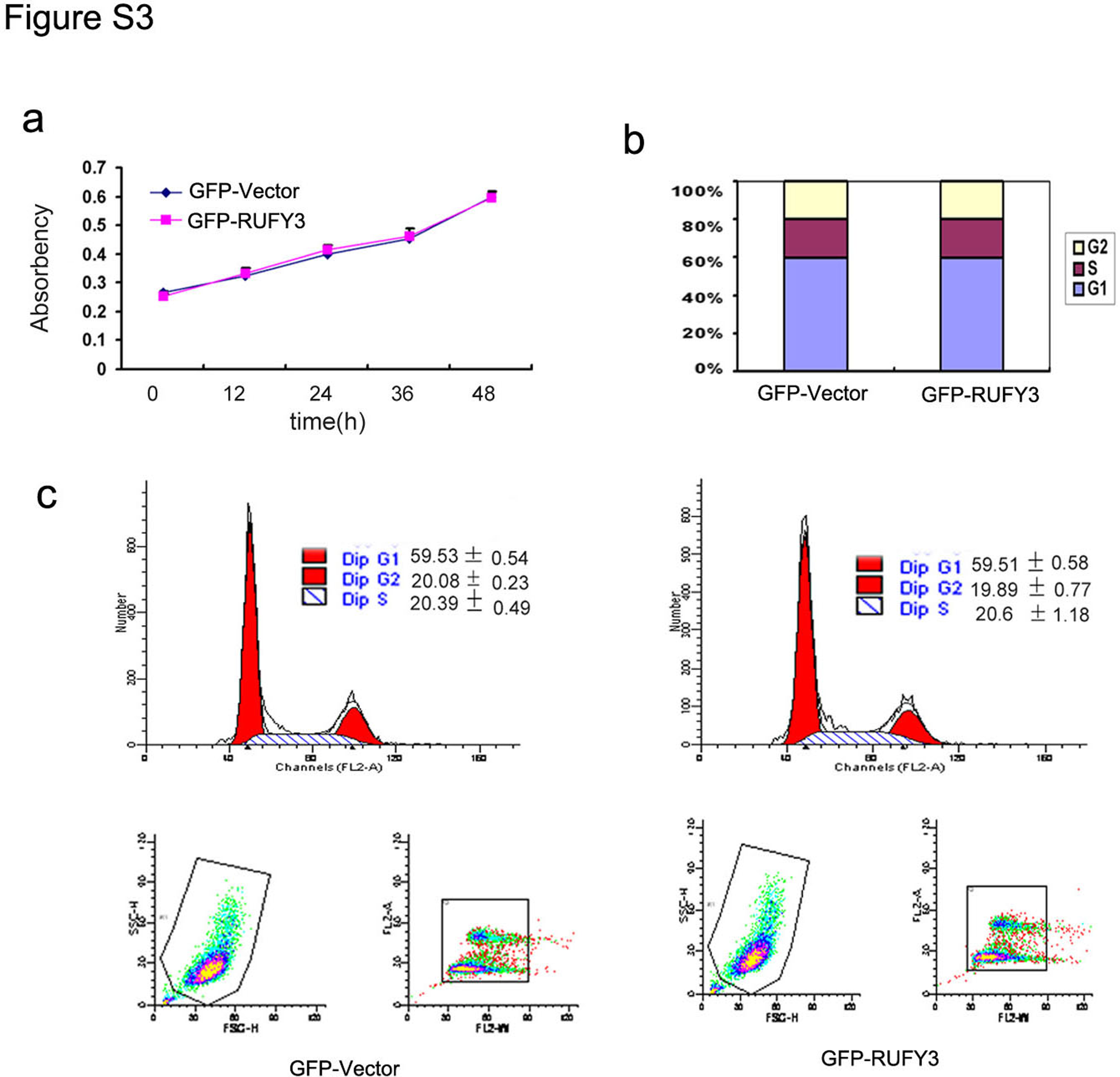

Supplement: Supplementary Figure 3 [file cddis201550x3.tif]

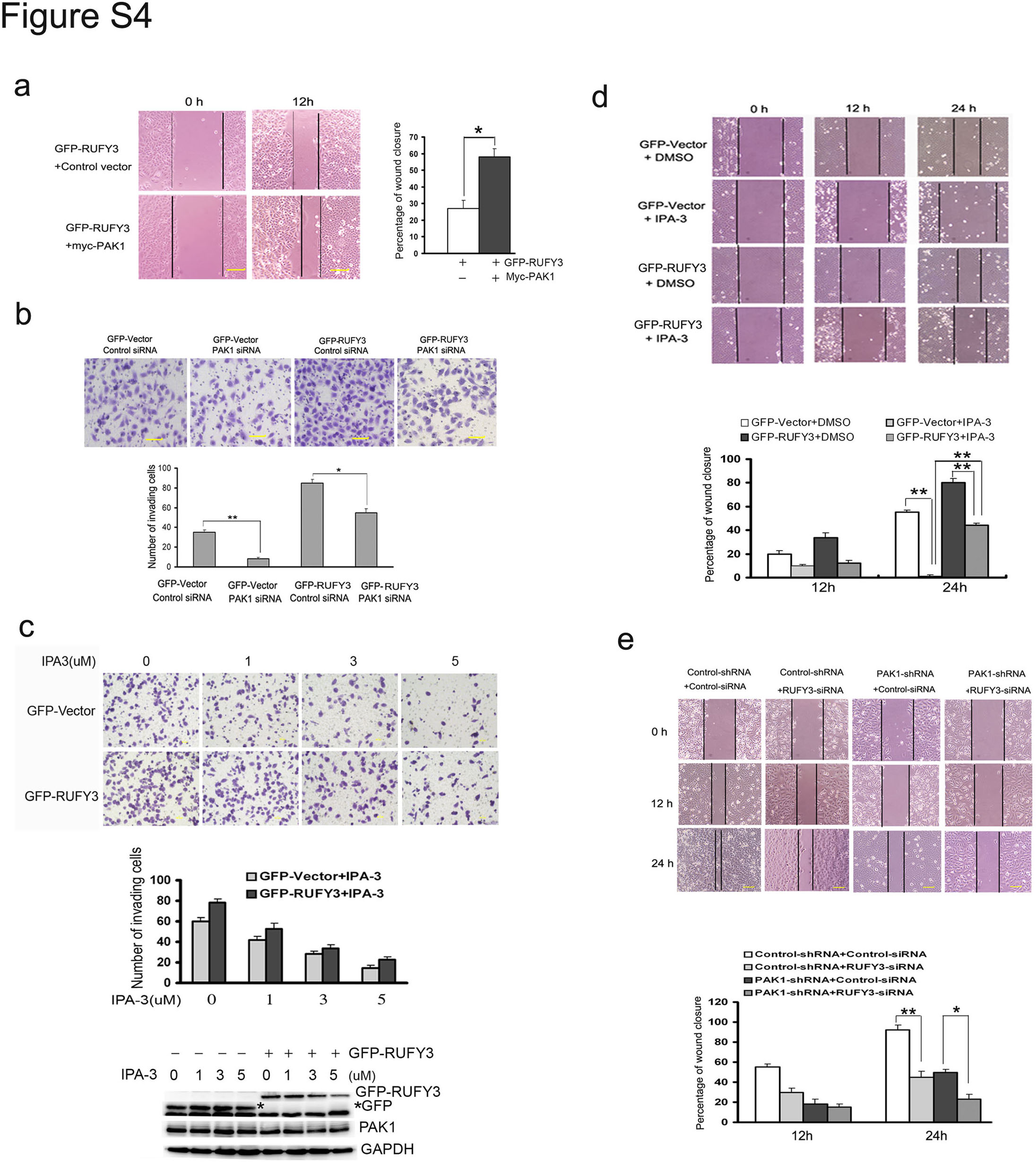

Supplement: Supplementary Figure 4 [file cddis201550x4.tif]

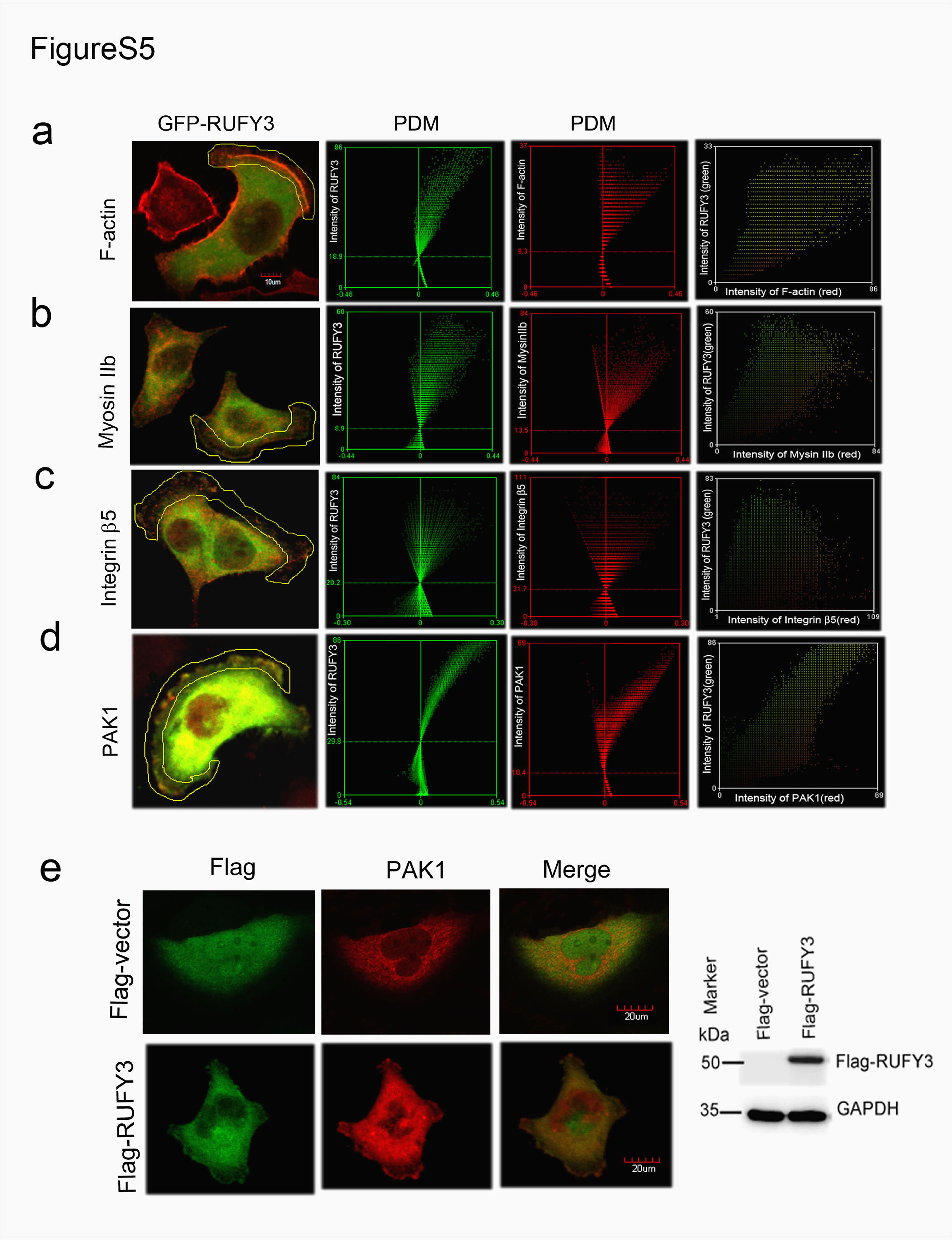

Supplement: Supplementary Figure 5 [file cddis201550x5.tif]
